# Tribological Behaviors of Super-Hard TiAlN Coatings Deposited by Filtered Cathode Vacuum Arc Deposition

**DOI:** 10.3390/ma15062236

**Published:** 2022-03-17

**Authors:** Zhiqiang Zhang, Lan Zhang, Heng Yuan, Menglin Qiu, Xu Zhang, Bin Liao, Fengshou Zhang, Xiaoping Ouyang

**Affiliations:** 1 Key Laboratory of Beam Technology of Ministry of Education, College of Nuclear Science and Technology, Beijing Normal University, Beijing 100875, China; zhiqiangzhang17@163.com (Z.Z.); lzhang1999@126.com (L.Z.); yuanheng1995@126.com (H.Y.); 11112020052@bnu.edu.cn (M.Q.); zhangxu@bnu.edu.cn (X.Z.); oyxp2003@aliyun.com (X.O.); 2 Beijing Academy of Science and Technology, Beijing 100875, China

**Keywords:** super-hardness, nanocrystalline TiAlN, microstructure, tribological behavior

## Abstract

High hardness improves the material’s load-bearing capacity, resulting in the enhancement of tribological properties. However, the high hardness is difficult to achieve for TiAlN coating due to the transformation of the close-packed structure from cubic to hexagonal and the increase in the grain size when the Al content is high. In the present study, the ultrahard TiAlN coatings (hardness > 40 GPa) are successfully developed by filtered cathodic vacuum arc technology to study the effect of nitrogen flux rate on tribological behaviors. The highest hardness of 46.39 GPa is obtained by tuning the nitrogen flux rate to achieve the regulation of Al content and the formation of nanocrystalline. The stable fcc TiAlN phase is formed via the solid-phase reaction under a high nitrogen concentration, and more aluminum atoms replace the titanium atoms in the (Ti, Al)N solid solution. The high Al content of the Ti_0.35_Al_0.65_N coating has a nanocrystalline structure and the average crystalline size is 16.52 nm. The TiAlN coating deposited at a nitrogen flux rate of 60 sccm exhibits the best properties of a combination of microhardness = 2972.91 Hv_0.5_, *H* = 46.39 GPa, *E* = 499.4 Gpa, ratio H/E* = 0.093 and ratio *H*^3^/E*^2^ = 0.403. Meanwhile, the TiAlN coating deposited at 60 sccm shows the lowest average friction coefficient of 0.43 and wear rate of 1.3 × 10^−7^ mm^3^ N^−1^ m^−1^ due to the best mechanical properties.

## 1. Introduction

TiN coatings are widely used in aerospace, mechanical transportation and chemical medical fields according to the combination of good properties in hardness, chemical inertness, wear resistance and corrosion resistance [1,2,3,4]. However, the low oxidation resistance makes TiN coatings fail because of the formation of brittle TiO_2_ when the temperature is high [5,6]. The existence of TiO_2_ showcases the potential risks of nanoparticle releases by coating or liquid dispersion and deteriorates environments and human health [7,8,9,10,11]. It is significant to inhibit the formation of TiO_2_ during the oxidation process to avoid nanoparticle release [12,13]. Adding elements, such as Al, Zr, Si, and Cr, is an effective way to improve the oxidation resistance [14,15,16,17,18]. Aluminum (Al) is one of the most attractive elements owing to its outstanding mechanical and physical properties, such as tribological properties and corrosion resistance [19,20].

TiAlN coatings could be prepared by ion beam sputter deposition [21], magnetron sputtering, arc ion plating and cathodic arc evaporation deposition. Moreover, cathodic arc evaporation technology is an effective method to produce less defects and high-density coatings due to the filtration of droplet particles in arc ion plating. Thus, the preparation quality of the coating is effectively guaranteed and the coating hardness can reach the super-hard level. M-R. Alhafian et al. deposited a Ti_50_Al_50_N monolayer coating with a hardness of 31.4 ± 0.7 GPa on carbide substrates by CAE, and found that high hardness contributes to improve wear resistance [22]. S. Sveen et al. studied the scratch adhesion of TiAlN coatings deposited on different substrates, such as high-speed steel, cemented carbide and PCBN. The results show that hardness is an essential parameter evaluating the mechanical performance, and usually represents the yield strength and wear resistance [23]. Ying Long et al. used cathodic arc evaporation (CAE) technology to prepare Ti_0.5_Al_0.5_N and Cr_0.3_Al_0.7_N coatings on silicon, and concluded that a Ti_0.5_Al_0.5_N coating has better flank wear resistance than a Cr_0.3_Al_0.7_N coating, owing to the higher hardness attributed to the smaller grain size [24]. The above literature pointed out that high hardness plays a crucial role in improving the coating’s property of resisting mechanical degradation. Nevertheless, the TiAlN coatings prepared by CAE exhibit medium hardness and the values are generally less than 40 GPa [22,23,24]. It is noted that the Al content ratio is a key factor affecting the hardness. The TiAlN coating shows the highest hardness of 31.4 GPa (3200 HV) when the Al/(Ti + Al) ratio is 60% [25]. Thus, adjusting the Al content is the key issue to obtain the high coating hardness. 

As we know, the existence of large particles in the CAE deposition process leads to a decrease in the strength and uniformity of the coatings and surface coarsening. Removing large particles can effectively enhance the quality of coatings. Filtered cathodic arc deposition (FCVA) could remove the neutral particles, macroparticles and droplets to obtain a smooth, hard and dense structure at a deposition process at a low temperature. It has the advantage of a high degree of ionization, high deposition and the easy control of deposition parameters. The regulation of composition, hardness, wear properties and morphology of the TiAlN coatings is achieved by the change of the cathode current, bias voltage on the sample, nitrogen gas pressure, magnetic filter elbow angle and magnetic intensity. Ivan A. Shulepov et al. prepared a high Al-content TiAlN coating on high-speed substrates by adjusting the arc current during the filtered vacuum arc deposition process and the hardness of the TiAlN coating achieved at 36 ± 4 GPa [26]. Cao et al. adjusted the substrate negative bias to prepare the bias-graded TiAlN coating on Ti_6_Al_4_V alloy substrates by FCVA at room temperature and the coating exhibited high hardness (32.08 GPa), adhesion and ideal toughness, along with excellent erosion resistance [27]. In addition, it was determined that nitride coatings and carbide coatings prepared by FCVA show excellent functional properties with high hardness, toughness and adhesion, indicating a significantly high mechanical stability, wear resistance and corrosion resistance [28,29,30,31]. Notably, FCVA technology is an energy-carrying ion beam deposition method. The atomic energy transfer can promote the rearrangement and diffusion of atoms in the micro region. Meanwhile, the instantaneous heating of microdomains inhibits the grain growth caused by long range diffusion, which helps to form fine nanocrystalline grains. Therefore, the FCVA is a flexible and an efficient process to produce a high mechanical performance, such as ultra-high hardness and good tribological resistance.

In this work, we use FCVA technology to deposit TiAlN coatings on 304L stainless steel and silicon wafer substrates, adjusting the nitrogen flux rates to regulate the Al content in the coating to obtain the ultra-high hardness. Additionally, the wear experiments were carried out to explore the correlations of the evolution of structure, adhesion and mechanical properties with tribological behaviors.

## 2. Experiment Detail

### 2.1. Coating Preparation

**Figure 1:**
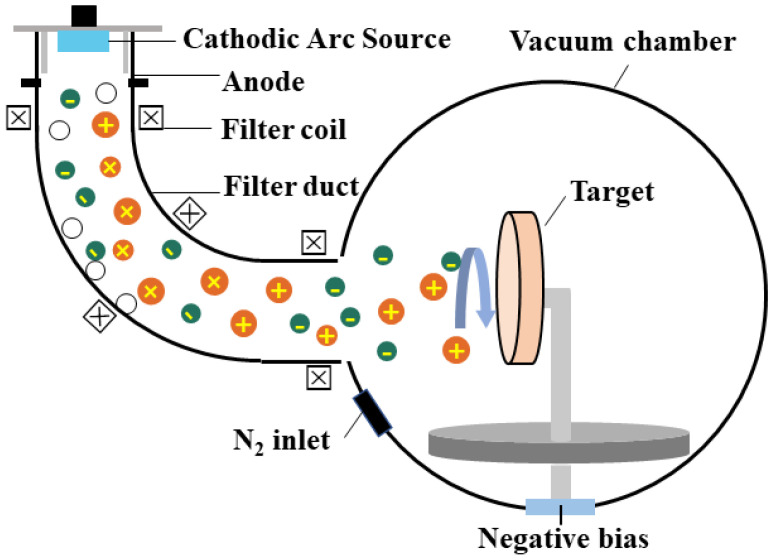
Schematic diagram of the FCVA deposition system.

In this work, the filtered cathodic vacuum arc deposition (FCVA) technique was selected to deposit TiAlN coatings on 304L stainless steel (20 mm × 20 mm × 2 mm) and silicon wafer substrates under various nitrogen flux rates. Prior to the deposition, the oil stains on the substrate surface were removed by ultrasonic cleaning in acetone and ethanol for 10 min sequentially. Figure 1 shows a schematic the diagram of the FCVA equipment. A Ti_0.33_Al_0.67_ target with a diameter of 100 mm was chosen as the cathode vacuum arc source, and the 90° magnetic filter duct was used in the system in order to ensure the deposition rate and filtration effect. The deposition details are showed in Table 1. The initial vacuum chamber was pumped to 4 × 10^−3^ Pa. Then, a high negative bias sputter process was used to activate and clean the sample surface. The process continued for 1 min under the bias voltages of −800 V, −600 V, and −400 V, respectively. During the deposition, a TiAl metal transition layer was firstly deposited on the substrates and the deposition time was kept at 10 min. Then, a nitrogen flux was introduced to and collided with the almost completely ionized Ti and Al plasma in the vacuum chamber. The entire TiAlN layer deposition process lasted for 80 min. The flux rate of the N_2_ gas was varied in five different conditions (0, 40, 50, and 60 sccm). Meanwhile, the work pressure was 3.3 × 10^−3^, 2.8 × 10^−2^, 3.7 × 10^−2^, 4.5 × 10^−2^ and 5.3 × 10^−2^ Pa. For each experiment, the chamber temperature was maintained at 300 °C. The detailed process deposition parameters of the TiAlN coatings are summarized in Table 1. 

### 2.2. Coating Characterization 

The surface morphology, cross-sectional microstructure and thickness of the coatings were observed by a field emission scanning electronic microscope (FESEM, HitachiS-4800, Hitachi Ltd., Tokyo, Japan). An energy disperse spectroscopy (EDS, EMAX-350, HORIBA, Ltd., Tokyo, Japan) system matched with the microscope used for the element analysis of the coatings. The transmission electron microscope (TEM, Philips/FEI CM200, Amsterdam, The Netherlands), was applied to further investigate the cross-sectional microstructure. The surface roughness was measured by an atomic force microscope (AFM, Tosca^TM^400, Melbourne, Austria). The tapping mode was used and the scanning range was 5 square microns. The Xpert Pro MPD X-ray diffraction (XRD, PANalytical, Almelo, The Netherlands) with Cu Kα radiation was used to determine the structure. The scanning rate was set to 2°/min and the testing ranged from 20° to 100°. The Scherer equation was used to calculate the crystallite size. The adhesion strength of the coating was qualitatively evaluated by a Rockwell tester equipped with a Rockwell C diamond indenter (tip radius R = 0.2 mm) with an applied load of 150 kg. a scratch test was carried out with an Anton Pear GmbH tester (Austria), equipped with a Rockwell C diamond tip (cone angle, 120°) to measure the adhesion quantitatively. The test was operated in progressive load test mode from 1 to 30 N in 5 mm. The Vickers indentation tester equipped with a diamond pyramid indenter was used to evaluate the microstructure and microhardness. The hardness (H) and elastic modulus (E) of TiAlN coatings were evaluated using an indentation tester (MML Nano Test P3, Tirol, Austria). The indentation depth was set to less than 10% of the total thickness of the coating and 9 positions were selected to calculate the average value of the hardness and elastic modulus. The H/E* and *H*^3^/E*^2^ ratio was used to qualitatively evaluate the evolution of the crack growth resistance, and E* was calculated using the formula of E* = *E*/(1 – *ν*^2^), where *ν* is the Poisson’s ratio of the coating material. The *ν* of TiAlN is taken equal 0.18 [32]. The residual stress of the TiAlN coatings was estimated by applying Stoney’s equation, where *E_s_*, *v_s_* and *t_s_* are the elastic modulus, Poisson’s ratio and thickness of the silicon wafer substrates, respectively, *t_f_* is the thickness of the coating, and *R*_1_ and *R*_0_ are the curvature radius of the substrate before and after depositing the coating, respectively.

$$\sigma =\frac{E_{s}t_{s}^{2}}{6\left(1-\nu_{s}\right)t_{f}}\left(\frac{1}{R_{1}}-\frac{1}{R_{0}}\right)$$

### 2.3. Friction Test

The wear property of the TiAlN coating was tested by a reciprocating wear test under dry conditions (MFTR4000, Lanzhou Institute of Chemical Physics, Lanzhou, China). A Si_3_N_4_ ball with a diameter of 6.5 mm was used as the friction counterpart. A normal load of 5 N was used in the test and the friction test lasted for 30 min. The sliding stroke and stroke frequency were set to 5 mm and 1 Hz. After the test, the wear rate was calculated by the wear profile obtained by the Taylor Hobson surface profilometer.

## 3. Results and Discussion

### 3.1. Coating Deposition Rate

**Figure 2:**
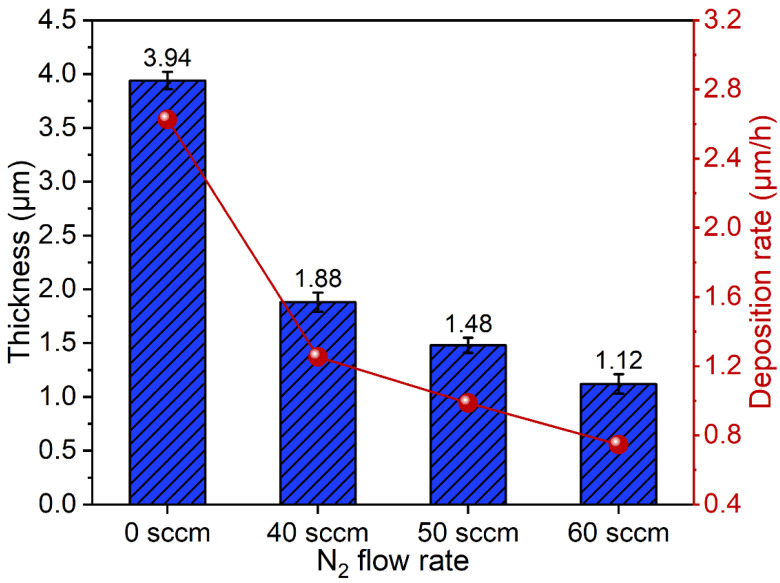
The coating thickness and deposition rate at different nitrogen flux rates.

The thickness and deposition rate of the TiAlN coatings are show in Figure 2. The thickness and deposition rate of TiAlN coatings exhibit decreasing trends, with an increase in the nitrogen flux rates. It can be observed that the deposition rate exhibits an obvious decrease when the nitrogen is introduced into the vacuum chamber. The reason for the decrease in deposition is mainly attributed to the target poisoning due to the fact that TiAl targets are partially covered by nitrides of Ti and Al, reduced from the emitting atoms and ions from targets. Similar results are reported in Refs. [33,34]. The maximum thickness of the TiAlN coating is up to 1.88 μm when the nitrogen flux rate is 40 sccm and the deposition rate reaches 1.25 μm/h. a fast deposition rate is one of the advantages of FCVA, which provides support for industrial application.

### 3.2. Structure and Elements

#### 3.2.1. Crystal Structure

**Figure 3:**
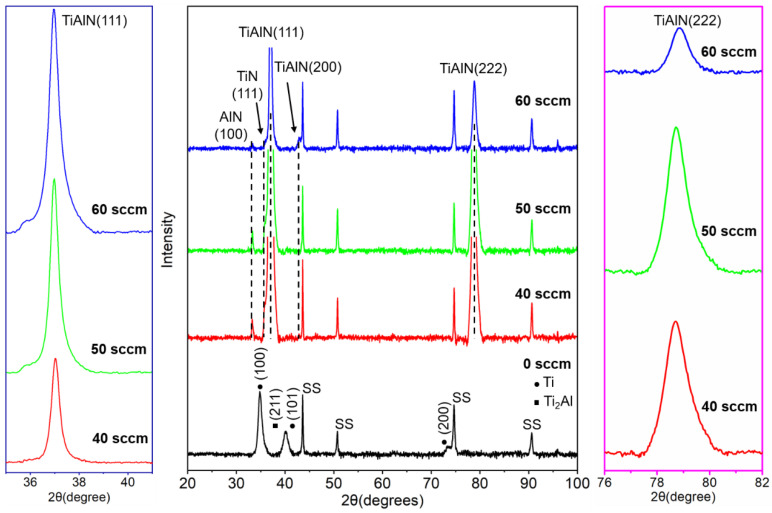
XRD patterns of TiAlN coatings and the magnification of the peak of TiAlN(111) and the peak of TiAlN(222).

The typical XRD spectra of TiAlN coatings with various nitrogen flux rates are presented in Figure 3. As shown in Figure 3, the TiAl coating (no N_2_) consists of Ti phase, and Ti_2_Al phase has both strong (100) and weak peaks. The intensity of (200) peaks in the TiAlN coatings is weak and the face-centered cubic (fcc) crystal structure with the preference of (111) (222) peaks can be observed. Additionally, there are weak peaks of c-TiN (111) (PDF-381420) and h-AlN (100) (PDF-251133), and the intensity is slight. Thus, the TiAlN coating consists of multi-phase structures, with a dominating fcc TiAlN phase and a small number of TiN and AlN phases. As the nitrogen flux rate increases, the intensity of strong (111) and (222) peaks gradually decreases. With the nitrogen flux rate of 60 sccm, the XRD pattern represents a smaller grain size and the half maximum (FWHM) increases. In addition, the intensity of the AlN (100) peak decreases with the increase in the nitrogen flux rate, indicating that the proportion of solid solution phase is higher when the nitrogen is sufficient. As we know, the formation of a stable TiAlN phase by the solid-phase reaction is due to the nitrogen concentration in the coatings and the process of deposition temperature [35,36]. In other words, the titanium atoms in the (Ti, Al)N solid solution are replaced by increasing amounts of aluminum atoms as the nitrogen flux rate increases. According to the solid solution strengthening mechanism, a high proportion of the solid solution phase is beneficial for improving the hardness [37]. The TiAlN coating prepared under a nitrogen flux rate of 60 sccm exhibits the highest hardness. In addition, the (111) and (222) diffraction peaks of TiAlN show a slight shift to a higher diffraction angle with the increase in the nitrogen flux rate. This is because the titanium atom is replaced by a smaller aluminum atom in the (Ti, Al)N solid solution, indicating that the presence of internal stress in the coating developed during the deposition [38]. 

The average FWHM values and crystallite size are shown in Table 2. When the nitrogen flux rate changed from 40 to 60 sccm, it can be observed that the FWHM values of TiAlN XRD (111) and (222) peaks increase from 0.326° and 0.656° to 0.398° and 0.979°, respectively. This phenomenon signifies the refinement of crystal grains in the coatings, which can result in a higher plastic deformation. M. Vashista et al. and M. Lindgen et al. reported that the FWHM value related to the plastic deformation of the material due to the relative increment in the dislocation density [39,40]. When the nitrogen flux rate increases, the titanium atom is replaced by a smaller aluminum atom in the (Ti, Al)N solid solution, resulting in the accumulation of defects, such as lattice distortion, which makes atoms easier to slip. Thus, the TiAlN coating prepared under a high nitrogen atmosphere has high plastic deformation and the FWHM value is the highest. The crystallite size is calculated by Scherrer’s equation as *D* = 0.9*λ*/(*β*cos *θ*), where λ is the wavelength of Cu/Kα radiation and is equivalent to 0.154 nm; β is the full width at half maximum (FWHM) and *θ* is the diffraction angle of the corresponding XRD peak. According to the XRD pattern image, we know that TiAlN coatings have a nanocrystalline structure, proved by the highly diffused nature of the peak. The results of the crystallite size further prove that all coatings are nanocrystalline and the grain size exhibits a decreasing trend. The TiAlN coating prepared under a nitrogen flux rate of 60 sccm shows the lowest average crystallite size with 16.5 ± 3.0 nm, contributing to the increased hardness and elastic modulus.

#### 3.2.2. Element Composition

The element composition of the coatings deposited at different nitrogen flux rates are listed in Table 3. When the nitrogen flux rate is 0 sccm, the Al content in the coatings deviates from its original 66.7% in the Al_67_Ti_33_ target due to the different ionized fraction, 50% for Al and 80% for Ti [25]. The Al/(Al + Ti) ratio increases from 31.19% to 65.56% when the nitrogen flux rate changes from 0 to 60 sccm. The following reasons may account for the changes in the Al content: first, the Al content is influenced by the charge state distributions of the ions in the ion flux. Al and Ti have different values for their average charge states, which are +1.7 and +2.1, respectively. This indicates that Ti exhibits a higher charge exchange cross-section than Al due to the high intensity of the Coulomb field. A higher amount of nitrogen increases the random collision direction of the ions. During this process, charge exchange may result in higher concentrations of lower-charged Al ions at the expense of high-charged Ti ions. Second, the Ti atoms in TiN are replaced by Al atoms to form a TiAlN solid solution, resulting in a decrease in the Ti content and an increase in the Al content. 

### 3.3. Morphology of Coatings

**Figure 4:**
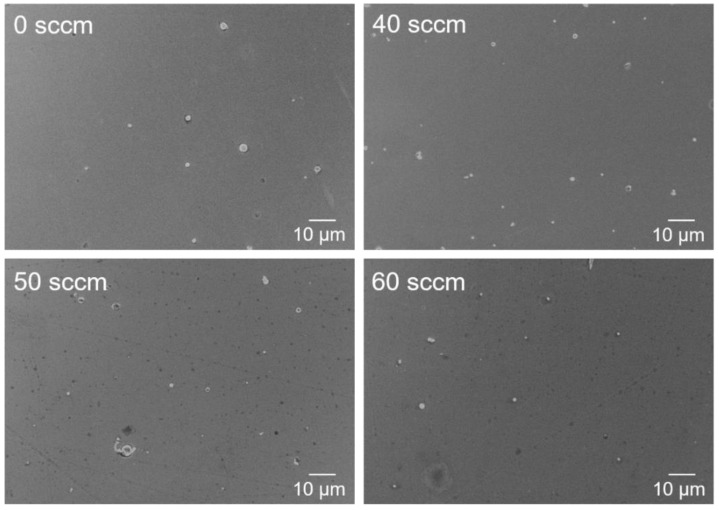
The surface of the TiAlN coatings prepared at different nitrogen flux rates.

**Figure 5:**
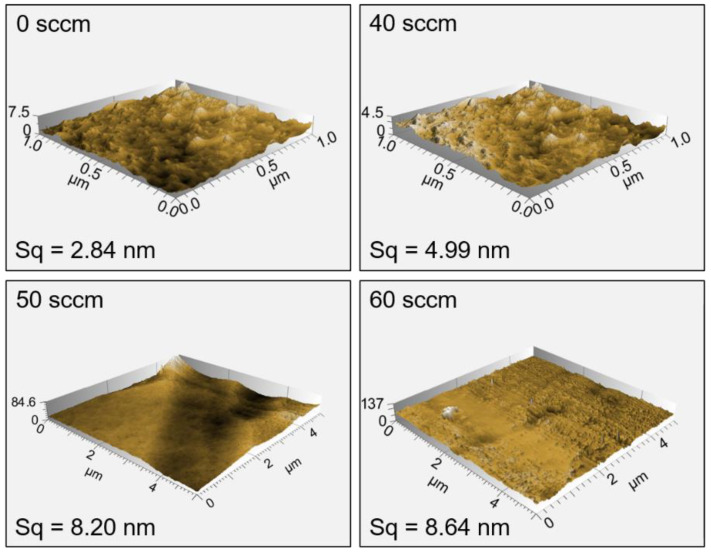
The roughness of the TiAlN coatings. Sq is the square roughness measured by AFM.

The surface morphology of the TiAlN coatings prepared under different nitrogen flux rates is presented in Figure 4. There are no obvious voids or cracks in the coatings, and they all exhibit a dense morphology. The surface of both coatings is flat and smooth, and few sub-micron-sized droplets could be observed. The quantity of sub-micron-sized droplets shows a slight increase when the nitrogen flux rates increase, which probably promotes the increased roughness. Fei Cai et al. reported that the macroparticle density of the AlTiN film gradually decreased with the increase in the nitrogen partial pressure due to a reduction in the momentum of the macroparticles by more random collisions with ions and atoms during the arc ion plating deposition process. The result opposes the one obtained in our work. The main reason is that the uncharged neutral droplets are not affected by the Lorentz force in the magnetic filter tube [31], and the number of droplets reaching the substrates is small. The roughness of the TiAlN coatings measured by AFM is shown in Figure 5. The results confirm that all TiAlN coatings have a smooth morphology and a change of roughness in a very small range of ~5.2 nm. The roughness increases from 2.84 nm to 8.64 nm when the nitrogen flux rate increases from 0 to 60 sccm. Both SEM surface morphology and AFM images indicate that FCVA can prepare dense TiAlN coatings with almost no droplets.

### 3.4. Cross-Sectional Morphologies

**Figure 6:**
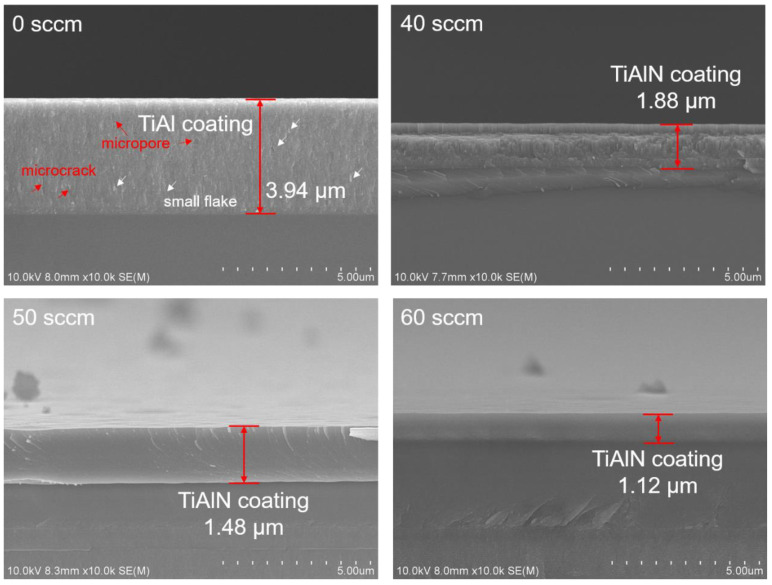
The cross-sectional images of the samples prepared under different nitrogen flux rates.

Figure 6 displays the cross-section morphology of TiAlN coatings deposited at different nitrogen flux rates. All TiAlN coatings show dense microstructures without obvious deposition defects, such as pores and cracks, and the coatings are tightly combined with the substrates. Flake-like and loose structures can be observed in the TiAl coating, indicating the low hardness in the mechanical properties. As show in Figure 6, nitrogen flux rates of 60 sccm, the white arrows denote small flakes, the red arrows denote microcracks and micropores, and microcracks can be clearly observed between the flakes. The coating deposited at the 40 sccm nitrogen flux rate exhibits a typical columnar structure, which grows outward from the coating–substrate interface and extends through the entire coating thickness. With the increasing nitrogen flux rate, the growth mode of the coating changed from columnar-to-near-equiaxed crystals. Therefore, the growth mode of the TiAlN coatings has an association with the nitrogen flux rate. The coating shows a columnar crystal structure at a low nitrogen flux rate and exhibits a near-equiaxed crystal structure at a high nitrogen flux rate. A similar result was reported in the literature [41].

**Figure 7:**
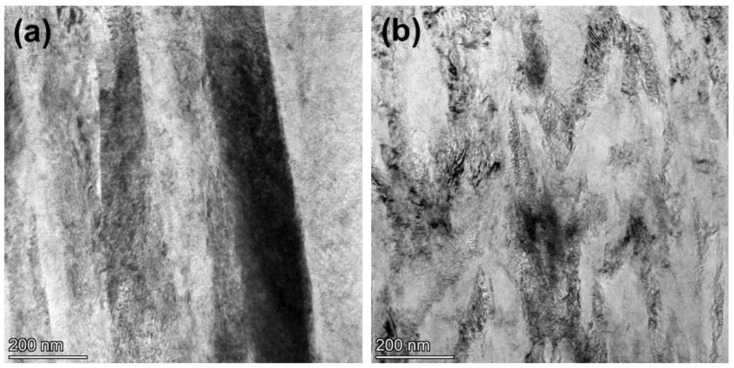
Cross section TEM micrographs of the TiAlN coating: nitrogen flux rate of (**a**) 40 sccm, (**b**) 60 sccm.

To characterize the coating’s structure at the highest resolution, TEM images of the cross-sectional area of the TiAlN coatings deposited at nitrogen flux rates of 40 and 60 sccm are performed in Figure 7a,b. A very high coating density can be observed, and no obvious holes are observed at the grain boundaries. The TiAlN coating prepared at a nitrogen flux rate of 40 sccm exhibits a columnar crystal structure, and the column’s width is nearly 100 nm and is fairly constant from the interlayer to the external surface. The finer columnar structures could be related to the higher deposition energy in the coating growth [22]. No clear boundary is observed in the TiAlN coating deposited at a nitrogen flux rate of 60 sccm, and the columnar crystal structure disappears. This result is also confirmed in the SEM cross-section morphological characterization. 

### 3.5. Mechanical Properties

**Figure 8:**
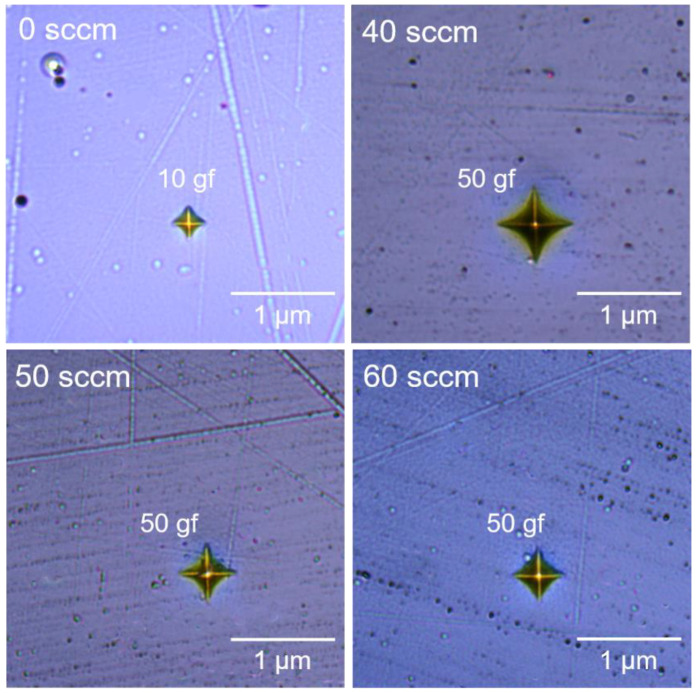
The optical micrograph of Vickers indentation of the TiAlN coatings deposited under various nitrogen flux rates.

Figure 8 shows the optical micrograph of the Vickers indentation of TiAlN coatings deposited at different nitrogen flux rates. The microstructure and microhardness of the TiAlN coatings are characterized by Vickers indentation with a load of 0.5 N. The load used for the TiAl coating was 0.1 N. The TiAl coating has the lowest Vickers microhardness of 318.68 HV_0.1_. When the nitrogen flux rate is 40 sccm, nitrogen, titanium and aluminum ions form a nitride coating and the Vickers hardness significantly increases to 2809.33 HV_0.5_. When the nitrogen flux rate increases from 50 to 60 sccm, the microhardness increases to 2837.34 HV_0.5_ and 2972.91 HV_0.5_. In the present study, the deposited TiAlN coatings have a hard coating. The generation of a covalent bond between nitrogen atoms and metal (Ti, Al) atoms could significantly enhance the surface hardness of metals and their alloys [42,43]. Meanwhile, no obvious microcracks are observed around and inside the indentation of the TiAlN coatings, indicating the high quality of those TiAlN coatings, which have high fracture toughness. 

**Figure 9:**
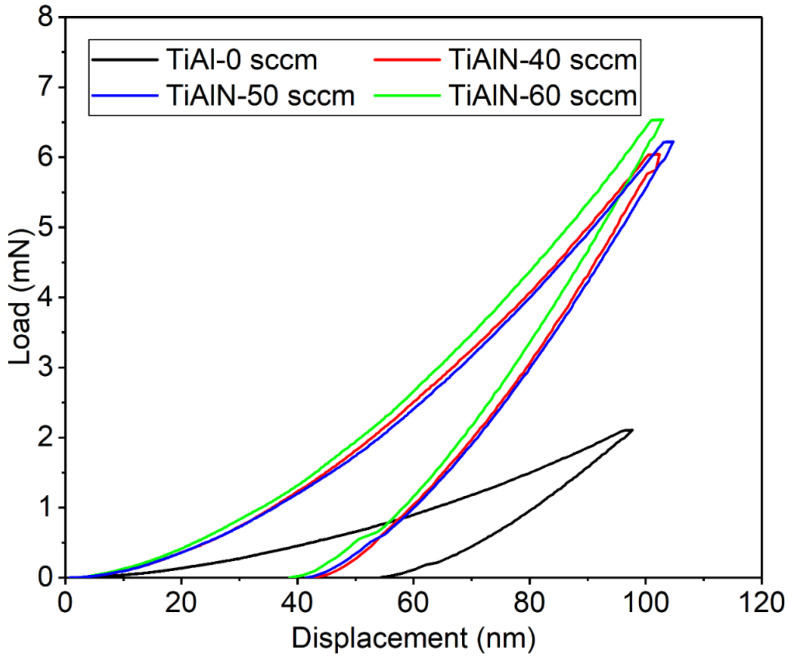
The loading–displacement curves for the TiAlN coatings deposited under different nitrogen flux rates.

The mechanical properties of the TiAlN coatings at different nitrogen flux rates can be characterized by hardness (*H*), Young’s modulus (*E*), H/E* and *H*^3^/E*^2^ values, and the details are shown in Table 4. The load–displacement curves are showed in Figure 9. The nanoindentation test was conducted at a maximum indenter penetration of 100 nm and the maximum load was less than 6.5 mN. It can be seen that the load-unloaded curves are smooth, without jumps and tears, indicating the high quality of the deposited TiAlN coatings. Similar to the results of the Vickers indentation test, the TiAl coating has the lowest hardness of 13.84 GPa. Compared with the reported TiAlN coatings deposited by HiPIMS, cathodic arc evaporation (CAE) and magnetron sputtering [22,44,45], the hardness values of all the TiAlN coatings presented in the present study are over 40 GPa, and those coatings could be considered as ultrahard. The hardness increases from 43.15 to 46.39 GPa as the nitrogen flux rate increases from 40 to 60 sccm. The nitrogen flux rate significantly affects the nano-hardness of the TiAlN coating. In addition, the change trend of Young’s modulus of the TiAlN coating is similar to the hardness. The coating exhibits the best mechanical properties at a nitrogen flux rate of 60 sccm. The coating hardness and Young’s modulus show the highest value of 46.39 GPa and 505.4 GPa, respectively. This can be attributed to the preferred orientation of TiAlN(111) and increased intensity of TiAlN(111). Cao et al. [41] reported that the hardness of TiAlN(111)-oriented coating is higher than the (200)-oriented coating, and this phenomenon can be explained from the relationship between the preferred orientation and the decomposed shear stress on the slip system [44]. Moreover, Table 3 shows that the Al content increases with the nitrogen flux rate, indicating more Al atoms instead of Ti atoms in the TiN lattice structure, which leads to an increase in internal stress because of the lattice distortion. At the same time, the dislocation pinning effect could be produced, and the sliding motion is hindered. Thus, the TiAlN coating obtains the highest value of hardness and Young’s modulus under the high nitrogen flux rate.

The H/E* and *H*^3^/E*^2^ ratios are related to the elastic strain resistance and plastic deformation resistance of the coating. High hardness and low elastic modulus can effectively improve the coating’s property of resisting mechanical degradation and failure toughness. In this paper, the H/E* ratio of TiAlN coatings slightly changes from 0.089 to 0.093, and the *H*^3^/E*^2^ ratio decreases from 0.375 to 0.361 GPa with an increase in the N_2_ flux rate from 40 sccm to 50 sccm; the nitrogen flux rate continues to increase to 60 sccm and the *H*^3^/E*^2^ ratio increases to 0.403 GPa. As we know, if the H/E* of the hard coating is greater than 0.1, the coating would have a high plastic deformation resistance and cracking resistance, resulting from the distribution of the load to a larger area [45,46]. High H/E*(≈0.1) and *H*^3^/E*^2^ (>0.35) ratios indicate that the TiAlN coatings in this work have good toughness while maintaining high hardness. High hardness, H/E* and *H*^3^/E*^2^ ratios contribute to improve the tribological properties [41]. From this point, the TiAlN coating deposited under 60 sccm exhibits good mechanical properties, with a combination of *H* = 46.39 GPa, *E* = 499.4 GPa, a ratio of H/E* = 0.093 and a ratio of *H*^3^/E*^2^ = 0.403; the tribological behaviors of the TiAlN coating prepared at a nitrogen flux rate of 60 sccm is expected to be the best. 

**Figure 10:**
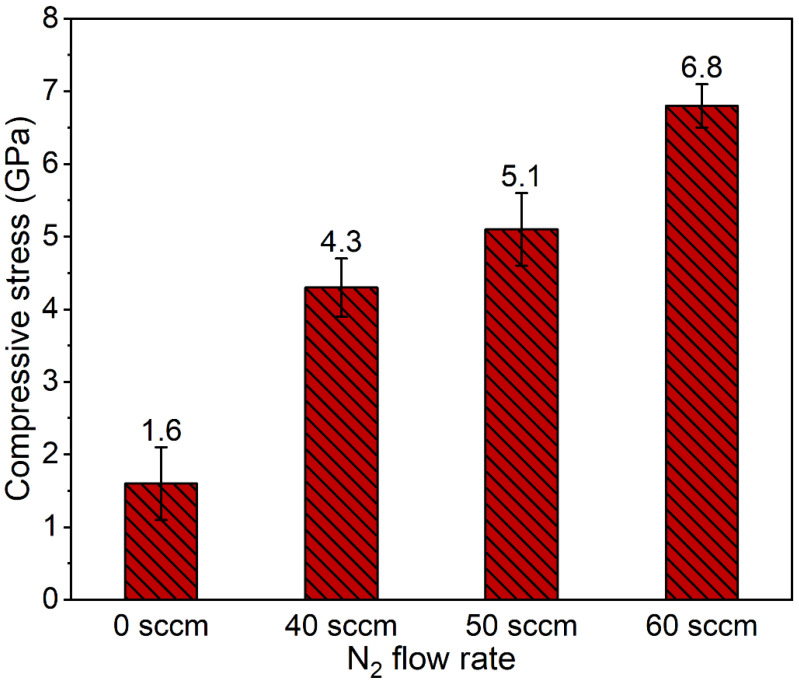
Residual stress of the different samples deposited using different nitrogen flow rates.

Figure 10 shows the coating stress obtained by the Stoney formula. All the coatings show compressive stress because of the intense ion peening effect induced by ion bombardment during the deposition process. As the nitrogen flux rate increases from 40 to 60 sccm, the compressive stress gradually increases from 4.3 GPa to 6.8 GPa due to the accumulation defects, such as lattice distortion. Residual stress has a significant effect on fracture toughness. Compressive stress can inhibit the generation and propagation of cracks, producing a pinning effect, which helps to enhance the fracture toughness of the coating. The TiAlN coating prepared under a nitrogen flux rate of 60 sccm has the best destruction toughness because of the high compressive stress. The result is consistent with the ratio of H/E* and *H*^3^/E*^2^.

### 3.6. Adhesion Strength

**Figure 11:**
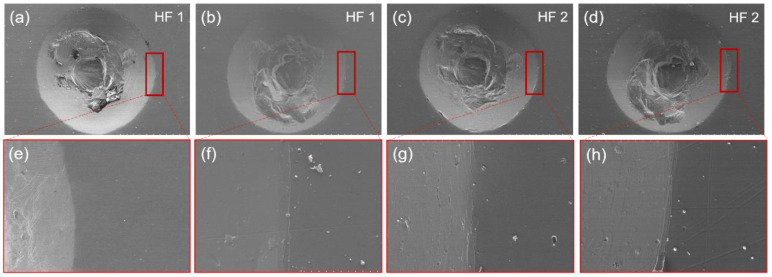
SEM morphology of Rockwell C indentation of the TiAlN coatings deposited at a nitrogen flux rate of (**a**,**e**) 0, (**b**,**f**) 40, (**c**,**g**) 50 and (**d**,**h**) 60 sccm.

The adhesion strength of the TiAlN coatings deposited at different nitrogen flux rates was assessed by Rockwell C indentations. HFl–HF4 defines an adequate adhesion, while HF5 and HF6 indicate an inadequate adhesion (HF is the German abbreviation for adhesion strength) [41]. Figure 11 presents the SEM morphology of Rockwell C indentation and the adhesion level of TiAlN coatings. As shown in Figure 11a–d, the plastic deformation is observed in the indentation center of all the coatings. It is attributed to the plastic deformation of 304L stainless steel. Previous studies have shown that soft substrate can generate a lot of plastic deformation when the standard load is applied [47,48]. At 0 sccm, there are no obvious cracks or exfoliation around the indentation in Figure 11a, because of the formation of the Ti and Ti_2_Al metal phase during deposition. Coatings prepared at a nitrogen flux of 40 sccm showed a good adhesion strength of HF1 with no observed cracks or delamination. With increasing the nitrogen flux rate to 50 and 60 sccm, some circumferential microcracks could be observed around the edges of the indentation in Figure 11c,d, but no delamination was observed. The adhesion strength can be defined as HF2. 

Three different critical loads, namely, Lc1, Lc2 and Lc3, are established to quantitatively evaluate the adhesion of the coating and substrate. When the first crack is formed along the scratch tracks and the substrate is not exposed, the corresponding load is marked as Lc1. Then, Lc2 is marked when the substrate is exposed along the borders of the track. Lc3 is the critical loading force for the complete spalling of coating.

**Figure 12:**
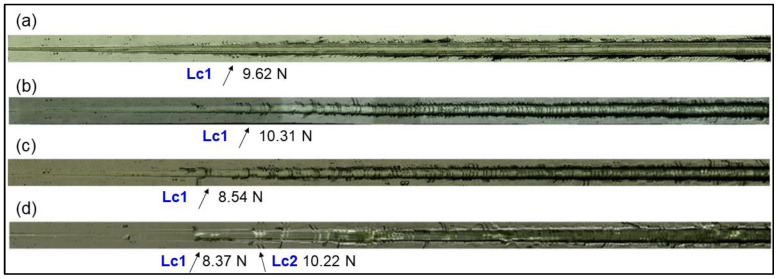
OM pictures of the scratch test for TiAlN coatings deposited under the nitrogen flow rates of 0 sccm (**a**), 40 sccm (**b**), 50 sccm (**c**) and 60 sccm (**d**).

Figure 12 shows the OM picture scratch and presents the critical loads. No complete exposure of the substrate can be observed in all the coatings and the Lc3 is not identified. At 0 sccm, the Lc1 is 9.62 N and Lc2 and Lc3 are not present. Lc1 decreases from 10.31 N to 8.37 N, while the nitrogen flux increases from 40 sccm to 60 sccm. There are slight variations for Lc1 at a different nitrogen flux rate, which is consistent with the adhesion strength of the Rockwell C indentation. The no peeling phenomenon indicates that those coatings have a good toughness to resist fractures and deformations. The TiAlN coatings exhibit a high adhesion to 304L stainless steel attributed to the dense structure and high mechanical property. 

### 3.7. Tribological Behavior

**Figure 13:**
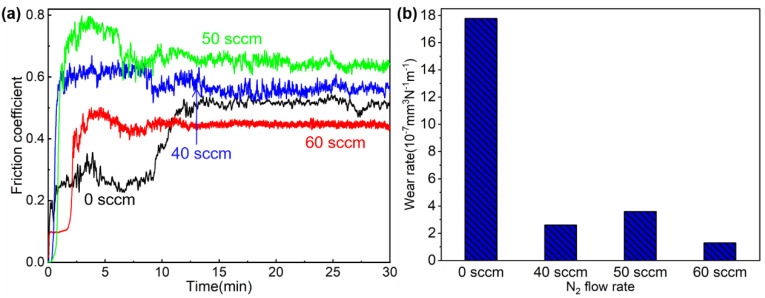
Tribological properties of different TiAlN samples: (**a**) friction coefficients, (**b**) wear rates.

The tribological behavior of TiAlN coating was studied by a reciprocating friction test in a dry friction environment. The corresponding friction pair is a Si_3_N_4_ sliding ball with the diameter of 6.5 mm. The load and reciprocating frequency are set 5 N and 1 Hz, respectively. The wear time is set at 30 min. Figure 13 presents the variations of the coefficient of friction of TiAlN coatings with the wear time. It can be seen that the friction curves of all the coatings are divided into two periods: early increase regime and steady regime. In the early increase regime, the real contact area grows in a short time, and grooves and abrasive particles are formed in the wear track, leading to a rapid increase in COF. After the COF reaches its highest value due to the interaction between abrasive particles and grooves, more and more abrasive particles serve as the bearing and separating medium, lowering the COF. When the quantity of abrasive particles and debris entering and exiting the wear track surface approaches a dynamic equilibrium, the COF enters a stable stage. It is noted that the TiAl coating shows a double increase–stable trend. The COF shows a rapid increase from 0 to 0.3 in 3 min, and then almost remains stable until the tenth minute. When the time shifts from 10 to 12 min, the COF sharply increases to 0.518 and retains a stable tendency. From the corresponding wear surface, a lot of grooves and debris are clearly observed owing to the plastic deformation. The wear mechanism is mainly dominated by adhesive wear and abrasive wear. Firstly, a large amount of metal debris is produced during the running period, which results in the rapid increase in COF. Meanwhile, the debris plays a lubricating role to stabilize the COF. However, with the increase in the sliding time, the accumulation of the debris is enhanced, which hinders the movement of the counterpart and further increases the COF, and, finally, stabilized at a value when a dynamic balance between the generation and overflow of wear debris is achieved. For all the TiAlN coatings, the friction coefficient rapidly increases and reaches the maximum value within 0–3 min, then begins to decrease, and then remains stable at 10 min until the end of the test. The coating with a nitrogen flux rate of 60 sccm has the lowest COF of 0.433, thanks to the high hardness and toughness. The average friction coefficient is about 0.6~0.8 [45,49,50]; thus, the friction performance of the super-hard, yet tough, TiAlN coating in this work was significantly improved.

From the results of the wear rate of the coatings, it can be concluded that the tribological performance is related to the gas flux rate for coating deposition. The TiAl coating shows the highest value of 17.8 × 10^−7^ mm^3^ N^−1^ m^−1^, with the extensive accumulation of debris as a result of the low hardness. When the nitrogen flux reaches 60 sccm, the average friction coefficient and wear rate have the lowest value of 0.433 and 1.3 × 10^−7^ mm^3^ N^−1^ m^−1^ owing to the highest hardness and H/E* and *H*^3^/E*^2^ ratios. As we know, the hardness plays a crucial role for the tribological performance of the hard coating. High hardness improves the material’s load-bearing capacity, resulting in the improvement of tribological properties. Moreover, the literature [49] reported that the combination of hardness and elastic modulus, that is H/E* and *H*^3^/E*^2^, also have an influence on the tribological performance. A higher hardness tends to reduce plastic strain when a load is applied to the coating surface, while a lower elastic modulus distributes a given load over a larger area, which reduces the maximum contact pressure. Therefore, high H/E* and *H*^3^/E*^2^ ratios present high cracking and plastic deformation resistance, meaning a better tribological performance.

**Figure 14:**
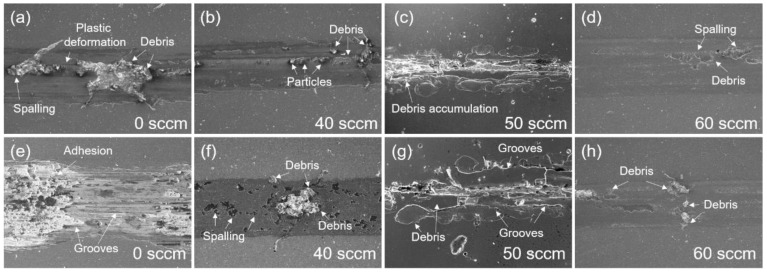
The SEM morphologies of the worn track of the TiAlN coatings deposited at different nitrogen flux rates. Wear track with a wear time of 3 min (**a**–**d**), wear track with a wear time of 30 min (**e**–**h**).

The SEM micrographs of the wear surface of the TiAlN coatings prepared at different nitrogen flux rates are showed in Figure 14. At the early stage of the wear test, it can be observed that the spalling, plastic deformation and debris accumulation begin to appear under the point load and are mainly distributed in the middle of the wear track, resulting in an increase in the friction coefficient. When the wear test is completed, there is an obvious adhesion phenomenon and grooves appear at the worn surface of the TiAl coating, indicating that the wear mechanism is mainly adhesion wear and abrasive. Meanwhile, the spalling and debris are distributed in the whole wear track section of the TiAlN coatings due to the transition from two-body wear to three-body wear, which is caused by the exfoliation of wear debris, meaning that the wear process has entered a stable stage. At 40 sccm, a large amount of debris was accumulated and the spalling area expanded, which indicates the occurrence of severe plastic deformation within the wear track [50]. In addition, the black wear track exhibits signs of oxidative wear [48]. It can be considered that the adhesive and oxidative wear mechanisms are dominant for the TiAlN coating deposited at 40 sccm. The wear track of the coating with a nitrogen flux rate of 50 sccm has large and obvious grooves. In addition, delamination and debris accumulation are also observed, indicating material transfer through the adhesive wear mechanism. Abrasive wear and severe adhesive wear were the main wear mechanisms of the coatings under a 50 sccm nitrogen flux condition. By increasing the nitrogen flux rate to 60 sccm, the wear track surface is smoother than it is for the other coatings, and a slight debris accumulation and spalling can be observed. This phenomenon can be explained by the highest H/E* and *H*^3^/E*^2^ ratios, which helps to minimize the ploughing impact.

## 4. Conclusions

The ultrahard TiAlN coatings (hardness > 40 GPa) were successfully developed on 304L stainless steel and silicon wafers by filtered cathodic vacuum arc technology. The effect of the nitrogen flux rate on the microstructure, and the mechanical and tribological behaviors was studied.

1. As the nitrogen flux rate increases, the deposition rate exhibits a decreasing trend, mainly attributed to target poisoning. The TiAlN coatings show a multi-phase structure dominated by a face-centered cubic (FCC) structure, with an increase in the intensity of a (111) peak, where the nitrogen flux rate increases from 40 to 60 sccm. The ratio of Al/(Al + Ti) increases from 31.19% to 65.56% owing to the formation of a solid solution.
2. The TiAlN coatings have a smooth morphology and a change in the roughness in a very small range of ~5.2 nm. All TiAlN coatings exhibit a compact microstructure with no apparent defects and tight bonding to the substrates. The growth mode of the coating from columnar-to-near-equiaxed crystals occurs with the increasing nitrogen flux rate.
3. With the increase in the nitrogen flux rate, the microhardness and nano-hardness show an increasing trend. The TiAlN coating prepared at a 60 sccm nitrogen flux rate exhibits the best mechanical properties.
4. When the nitrogen flux rate increases from 40 sccm to 60 sccm, the average friction coefficient first increases and then decreases, which is opposite to the changes of the values of the H/E* and *H*^3^/E*^2^ ratios. Coatings deposited at a 60 sccm nitrogen flux rate have the best tribological properties due to the high hardness and H/E* and *H*^3^/E*^2^ ratios.

The technique, results, and mechanisms described herein provide insights and guidance on how to prepare a super-hard TiAlN coating by FCVA. The application of TiAlN coatings in industrial fields should be further expanded.

## Figures and Tables

**Table 1:** Detailed deposition parameters of the TiAlN coatings.

Parameters	Values
Target	Ti_0.33_Al_0.67_
Positive bias voltage (V)	24
Cathode current (A)	100
Filter coil current (A)	2.6
Bias voltage (V)	−100
Duty ratio (%)	90.5
Background pressure (Pa)	4.5 × 10^−3^
Deposition time (min)	10 + 80
Substrate temperature (°C)	300
N_2_ (sccm)	0/40/50/60

**Table 2:** FWHM and crystallite size of the TiAlN coatings.

N_2_ Flux Rate (sccm)	TiAlN(111) (36.99° to 37.05°)		TiAlN(222) (78.75° to 78.86°)	
	FWHM (°)	Crystallite Size (nm)	FWHM (°)	Crystalline Size (nm)
40	0.326 ± 0.042	26.9 ± 2.3	0.656 ± 0.028	16.4 ± 3.1
50	0.353 ± 0.038	24.9 ± 2.7	0.673 ± 0.043	16.0 ± 3.5
60	0.398 ± 0.036	22.1 ± 2.1	0.979 ± 0.019	10.9 ± 3.8

**Table 3:** Chemical composition tested by the EDS of TiAlN coatings.

N_2_ Flux Rate (sccm)	Ti (at.%)	Al (at.%)	N (at.%)	Al/Al + Ti
0	68.8 ± 0.3	31.2 ± 0.3	0	31.19%
40	20.1 ± 0.2	28.3 ± 0.2	51.6 ± 0.2	58.46%
50	18.5 ± 0.3	29.6 ± 0.3	51.9 ± 0.2	61.54%
60	16.5 ± 0.2	31.3 ± 0.3	52.2 ± 0.3	65.56%

**Table 4:** Mechanical properties of the TiAlN coatings deposited at different nitrogen flux rates.

N_2_ Flux Rate (sccm)	*H*/(GPa)	*E*/(GPa)	*H*^3^/E*^2^ (GPa)	H/E*
0	13.8 ± 2.6	173.7 ± 7.0	0.088	0.079
40	43.2 ± 1.7	494.2 ± 12.8	0.375	0.090
50	44.8 ± 1.4	499.8 ± 11.2	0.361	0.089
60	46.4 ± 1.8	505. 5 ± 13.3	0.403	0.093

## Data Availability

The data used to support the findings of this study are available from the corresponding author upon request.
